# Sub-10 nm colloidal lithography for circuit-integrated spin-photo-electronic devices

**DOI:** 10.3762/bjnano.3.98

**Published:** 2012-12-19

**Authors:** Adrian Iovan, Marco Fischer, Roberto Lo Conte, Vladislav Korenivski

**Affiliations:** 1 Nanostructure Physics, Royal Institute of Technology, 10691 Stockholm, Sweden

**Keywords:** magnetic point contact arrays, spin laser, sub-10 nm colloidal lithography

## Abstract

Patterning of materials at sub-10 nm dimensions is at the forefront of nanotechnology and employs techniques of various complexity, efficiency, areal scale, and cost. Colloid-based patterning is known to be capable of producing individual sub-10 nm objects. However, ordered, large-area nano-arrays, fully integrated into photonic or electronic devices have remained a challenging task. In this work, we extend the practice of colloidal lithography to producing large-area sub-10 nm point-contact arrays and demonstrate their circuit integration into spin-photo-electronic devices. The reported nanofabrication method should have broad application areas in nanotechnology as it allows ballistic-injection devices, even for metallic materials with relatively short characteristic relaxation lengths.

## Introduction

Colloidal lithography [1] is a method to reproduce patterns in a variety of natural systems and is used more and more as an efficient fabrication tool in bio-, opto-, and nanotechnology. Nanoparticles in the colloid are made to form a mask on a given material surface, which can then be transferred by etching into nanostructures of various sizes, shapes, and patterns [2–3]. Such nanostructures can be used in biology for detecting proteins [4] and DNA [5–6], and for producing artificial crystals in photonics [7–8] and gigahertz oscillators in spin-electronics [9–14]. Scaling of colloidal patterning down to 10 nm and below, dimensions comparable or smaller than the main relaxation lengths in the relevant materials, including metals, is expected to enable a variety of new ballistic transport and photonic devices, such as spin-flip terahertz lasers [15]. In this work we extend the practice of colloidal lithography to produce large-area, near-ballistic-injection, sub-10 nm point-contact arrays and demonstrate their integration into spin-photo-electronic devices.

Electron-beam and focused-ion-beam techniques are typically limited to feature sizes of tens of nanometres, if the features are to be well defined, and are rather inefficient for large-area nanopatterning since both methods employ series point-by-point pattern transfer. The two promising techniques of nano-imprint lithography [16–17] and extreme-ultraviolet interference lithography [18] do indeed open sub-10 nm nanostructures for exploration. The instrumentation required, however, can in many cases be of great complexity and cost. Recently, membranes of nano-porous anodic aluminium oxide [19] were shown to scale to sub-10 nm dimensions and potentially compete with the above advanced lithographic techniques at this scale. Another potential alternative for sub-10 nm patterning is colloidal lithography, which is very attractive at larger dimensions due to its ease of use and low cost. Colloid-based patterning is known to be capable of producing individual sub-10 nm objects. However, ordered large-area nano-arrays fully integrated into photonic or electronic devices have not been demonstrated by using colloidal lithography. In this work we use a self-assembled monolayer of polystyrene nanoparticles, reduced in size by an isotropic etching process [20], which we scale to sub-10 nm feature sizes with large-area coverage in a well-defined hexagonal lattice and full integration for electrical circuit biasing and read out. We demonstrate the fabrication technique using spin-torque and spin-flip photoemission material combinations, considered promising for gigahertz oscillators and terahertz lasers.

## Results and Discussion

### Self-assembled monolayer of nanoparticles

The most widely used colloidal lithography medium is polystyrene nanoparticles in aqueous solution. Such colloidal solutions are commercially available with a variety of concentrations and particle sizes [21]. We used a range of diameters (down to 40 nm) and found the most consistent results in terms of self-assembly for 200 nm diameter and 2% particle concentration. Different methods of forming a monolayer of colloidal particles on a surface exist [22]. We found the spinning of the polystyrene colloidal water solution to yield good results. In calibrating the speed and duration of the spinning we aimed at forming the largest-area continuous monolayer possible. Thus, spinning in three stages, 500 rpm for 10 s, 1000 rpm for 30 s, and 2000 rpm for 10 s, facilitated self-assembly and yielded continuous nanoparticle monolayers of hundreds of micrometres in area (see Experimental for details). This was sufficient for our purposes to demonstrate a wide range of integrated device sizes.

**Figure 1:**
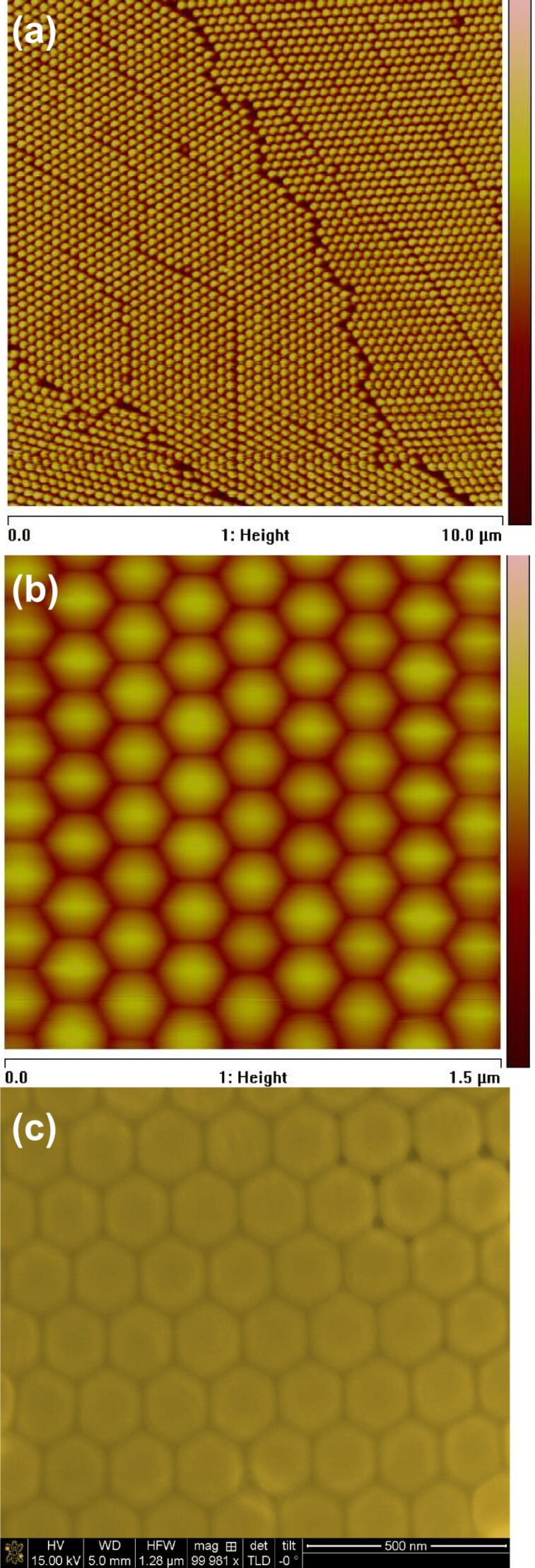
Tapping-mode atomic force microscopy images of a typical monolayer, with the particle diameter (and the inter-particle distance) of 200 nm on (a) a large scale and (b) a small scale. Scanning electron microscopy image of the sample (c) shows that the nanoparticle array has a nearly perfect close-packed hexagonal lattice.

Figure 1a and b show the tapping-mode atomic force microscopy (AFM) images of a typical monolayer, with the particle diameter (and the interparticle distance) of 200 nm. The monolayer film is of good quality, with only minor defects on the large scale (Figure 1a). On the small scale (Figure 1b) the lattice is clearly hexagonal. The scanning electron microscopy (SEM) image of the sample in Figure 1c shows that the nanoparticle array has a nearly perfect close-packed hexagonal lattice. The dispersion in the particle size at 200 nm diameter is small (approximately 1%), which favours the translation of the hexagonal pattern over large areas, i.e., hundreds of microns in the case of our optimized self-assembly process.

### Downscaling to sub-10 nm

**Figure 2:**
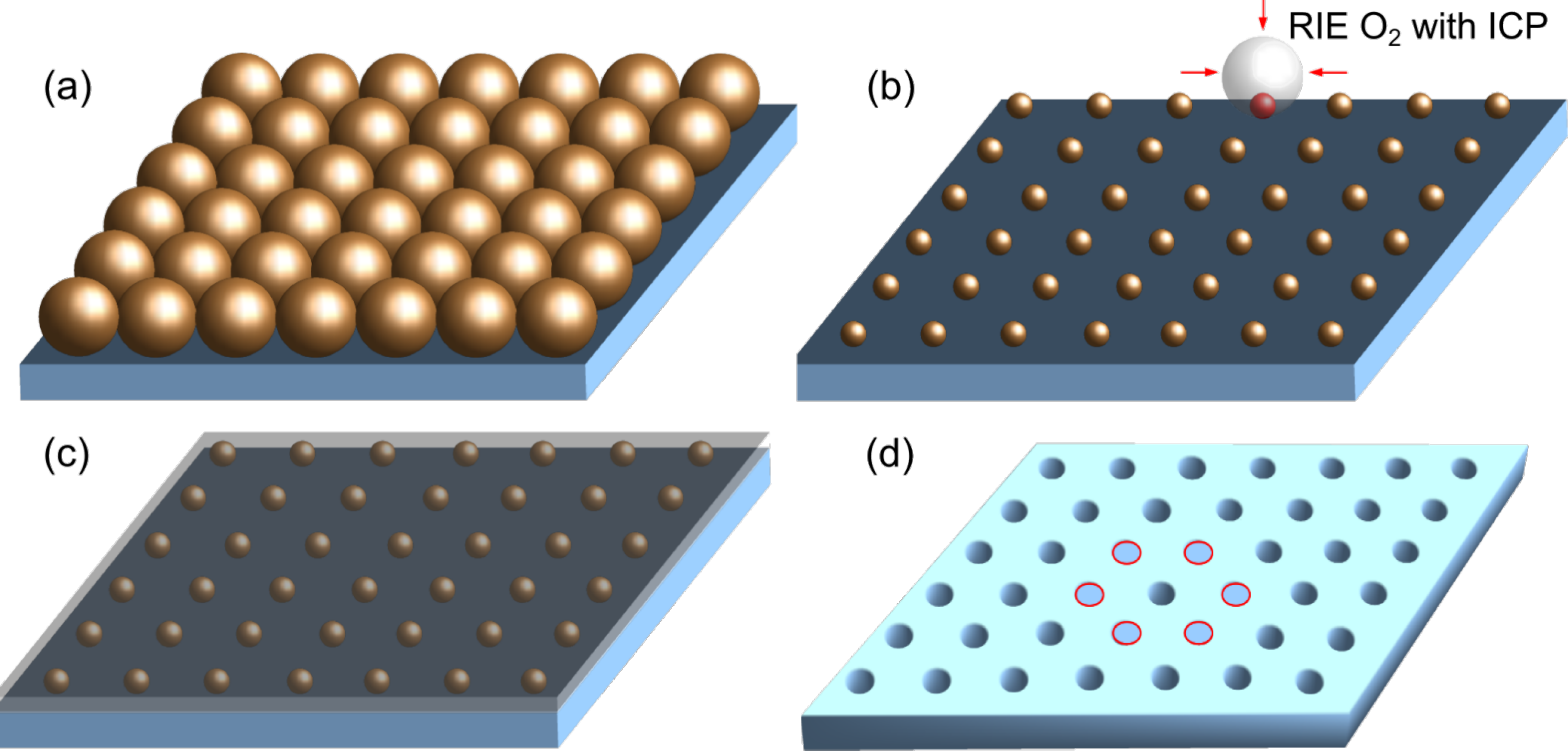
Illustration of the four steps in down-scaling of the particles of a polystyrene monolayer to the 10-nm range, which is to later serve as a nano-array mask. (a) Forming a self-assembled hexagonal-close-packed monolayer. (b) Reactive oxygen plasma with ICP is used to reduce the size of the particles. (c) A reinforcing layer of aluminum is deposited to serve as a hard mask. (d) A lift-off of the particles by etching completes the fabrication of the nanomask.

Our process of down scaling the particles of the polystyrene monolayer to the 10-nm range consists of four main steps, illustrated in Figure 2. Once the monolayer is formed (Figure 2a), reactive oxygen plasma is used to reduce the size of the particles (Figure 2b). When the desired particle diameter is reached, a reinforcing layer of aluminium is deposited, as shown in Figure 2c, which in the later process stages acts as a hard mask. A lift-off of the particles by etching completes the fabrication of the nanomask, as illustrated in Figure 2d. The very sensitive process step of the downscaling of the particles is achieved by reactive plasma etching, which must be done in a very clean chamber [23] in order to have a uniform reduction in the particle size across the large-area array. The final particle diameter is found to be a smooth function of the etching time, so the feature size of the nanomask can be controlled rather precisely. The typical etching power used is relatively low (50 W) to avoid potential disruptive etching at higher power. The key process detail, that we found to be critical for achieving sub-10 nm resolution, is a superimposed inductively coupled plasma (ICP) of relatively high power (250 W), which increases the ionization in the chamber, translating into a more isotropic reduction in the particle size. A previous study of isotropic etching for nanosizing of polystyrene particles has shown the high capability of colloidal lithography [20]. According to this study the sample temperature has a strong influence on the etching process and can be critical for the uniformity of the etching. In our process we keep the sample temperature constant at near room temperature (30 °C) using a liquid-nitrogen cooling line. The polystyrene particles remain nearly spherical during the process, even as their size is reduced by more than an order of magnitude. The oxygen pressure was found to be optimum at 40 mTorr.

**Figure 3:**
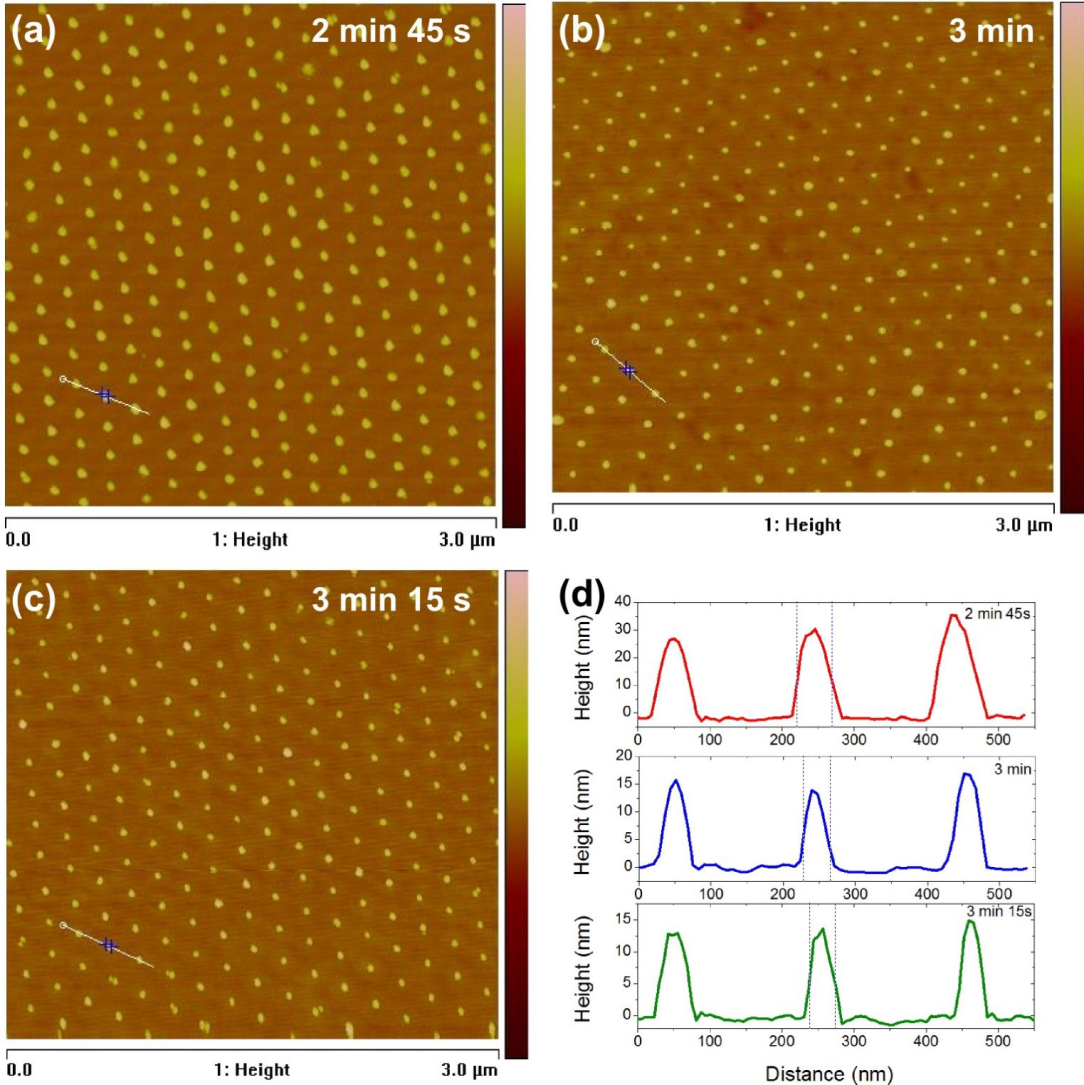
Polystyrene nanoparticles monolayer after plasma-ICP etching for (a) 165, (b) 180, and (c) 195 seconds. (d) AFM height profiles of the three samples shown in (a–c): raw data, without deconvolution.

A typical monolayer after the etching procedure is shown in Figure 3, imaged by AFM for etching quality, surface topography, and particle size. The height of the particles is measured accurately, but not the diameter, since the convolution of a small particle and the tip produces a width distortion. Keeping all the process parameters constant and varying only the ICP etching time, we reproduce the general result of the previous studies [2,20] of a gradual reduction in the particle size. In our case, the particles remain well attached to the substrate and form a well-defined hcp pattern down to the smallest dimensions of 10–15 nm, as discussed below.

Figure 3a–c, show the particle monolayer after etching for 165, 180, and 195 seconds, respectively. The key advance made in this work, compared to the results on colloidal lithography reported to date, is that our modified process scales to sub-10 nm dimensions. For example, for the etching time of 2 min 45 s (Figure 3a) the particle diameter is reduced to 10–15 nm (measured by AFM with particle–tip deconvolution). For 3 min etching time the average diameter is below 10 nm (Figure 3b). Etching for 3 min 15 s reduces the average size further but induces some perturbations. Already at 3 min 30 s etching time the monolayer is significantly over-etched and the pattern is partly removed. The true size of the particles can be estimated by using AFM traces, such as those shown in Figure 3d. The size of the particles here is smaller than the actual curvature of the AFM tip, determined from scanning calibration samples to be approximately 50 nm. Therefore, a deconvolution procedure was used to obtain the particle sizes stated above, which compare well with those obtained by SEM (see below). The AFM height is a more direct measurement and shows approximately 30 nm for 2 min 45 s and 15 nm for 3 min etching time. Figure 3 thus illustrates the fine control of the particle size at ≈10 nm by varying the plasma etching time.

The technological viability of the obtained polystyrene nanoparticle array depends on the ability to transfer it into a reliable mask to be used in subsequent nanodevice integration. The most straightforward approach, used widely in the literature for patterns of larger particle size, would be to directly etch the underlying substrate (e.g., SiO_2_, Si, or Au) using the particle array as the mask. Our extensive tests showed, however, that the particle-mask itself is significantly modified during this process, which makes the pattern transfer at the desired 10 nm diameter range essentially impossible. We therefore developed an additional lift-off process step to reinforce the mask. It includes a deposition by e-beam evaporation of an Al metal layer onto the etched particle array, with a subsequent lift-off step to remove the polystyrene particles, and yields the hard mask illustrated in Figure 2d. This transferred the particle pattern into a hole mask of Al, with slightly larger particle sizes, 15–30 nm, rather than the smallest particles we could achieve (≈10 nm). The thickness of the Al reinforcing layer (e.g., 15 nm) was selected to be less than the particle size used, such that the subsequent oxygen-RIE step could reach and remove polystyrene through the thinned Al at the sides of the particles. It was found that the Al hard mask effectively reduced the feature size, possibly due to some shadow-filling and/or particle-shape modification during Al deposition. We found this mask-transfer process to reliably yield hole-masks in the ≈10 nm range, as verified by SEM, AFM, and transport data.

A successful and stable lift-off process at these small length scales was found to be reactive ion etching (RIE) with oxygen, in which the polystyrene particles are first etched predominantly from the sides, where the Al film is much thinner due to the shadowing effect of the e-beam coating of the polystyrene spheres. During this RIE etching step the Al film surface oxidizes and forms a hard mask for subsequent ion milling. After a 5 min Ar-plasma etch to remove surface residue, 7 min long ion milling etches through the 10 nm thick Au layer and slightly into the SiO_2_ substrate, thus transferring the hexagonal pattern of sub-10 nm polystyrene particles into sub-10 nm pattern of holes in Au on SiO_2_. The Al-oxide layer acts as the hard mask in this process. The Au under-layer for the polystyrene-particle monolayer was used in the process from the beginning, but was later found to be not critical for the process, and similar results were obtained without this layer (between SiO_2_ and Al).

**Figure 4:**
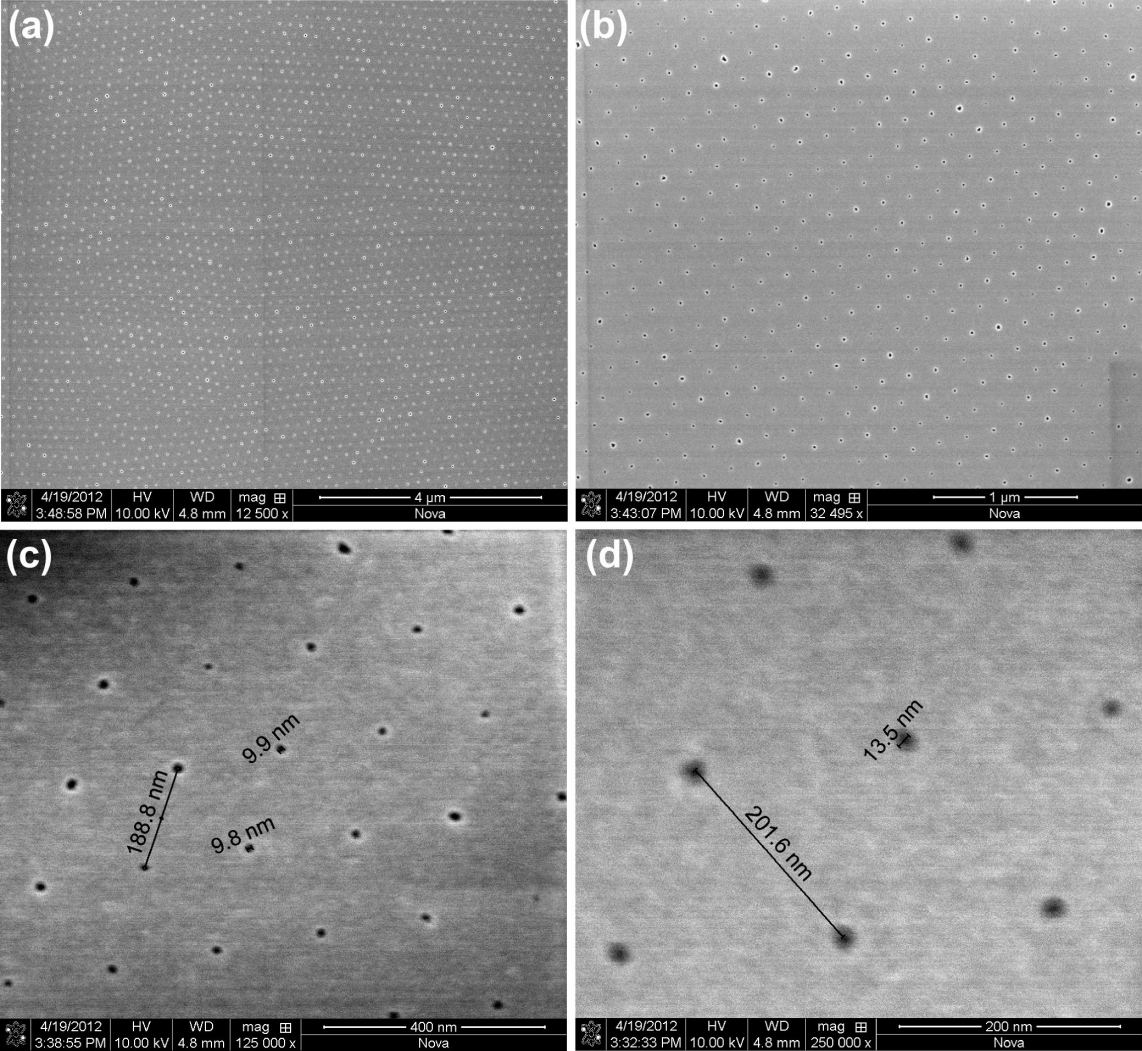
SEM images of a typical colloidal-monolayer mask, shown on four scales in (a–d). The average interdot distance is 200 nm (c,d), corresponding to the original polystyrene particle diameter. The average dot size is variable by adjustment of the ICP etching time, and reaches down the sub-10 nm range (c). (d) Shows a mask close-up with a nearly perfect hexagonal-close-packed pattern, with a lattice constant of 200 nm and a hole diameter of 13 nm. (a–d) correspond to different sections of the same mask.

Figure 4 shows SEM images of a typical nanohole array mask. Long-range order is maintained over the micrometre range, as shown in Figure 4a and b. The process was repeated many times and showed good reproducibility. The average interdot distance is 200 nm, corresponding to the original particle diameter (Figure 4c and d) and the average hole size reaches down to the sub-10 nm range. The SEM data is well calibrated and confirms the deconvoluted AFM data discussed above as regards the morphology of the array and the size of the particles, throughout the process.

### Device integration

Having developed a reliable process for producing nano-dot array masks scalable to sub-10 nm dimensions, we next demonstrate their integration into advanced spin-photo-electronic devices, such as new types of nano-oscillators [9–14] and the newly proposed lasers [15]. The 1–10 nm scale is particularly interesting as it enables devices based on nonequilibrium injection, even for metals, due to the single-dot size being comparable or smaller than the characteristic relaxation length scales in the material. Fabricating and integrating sub-10 nm dot arrays into circuit-driven devices is a nontrivial task for any patterning technique (see Introduction) and, to our knowledge, has not been demonstrated to date.

For the spin-laser device of [15], for example, the bottom electrode must be thick to serve as an efficient electron and phonon bath under high-current injection. We take that into account in the design and start the structure with a sputter-deposited tri-layer of Al(180 nm)/SiO_2_(15 nm)/Au(10 nm) onto a Si/SiO_2_(500 nm) substrate. The bottom electrode, later to serve as one side of a 10–100 µm range optical resonator, is patterned by using standard optical lithography (see Experimental for details).

The sample is then cleaned by oxygen RIE for 2 min, which makes the surface hydrophilic, and covered with 2–3 drops of the colloid solution forming the polystyrene particle monolayer during the above spinning sequence. The particles are scaled down using the multistep process detailed above to form a nanohole array mask on the bottom electrode surface. Plasma RIE with CF_4_ is used for 2 min for making the contact through the 15 nm thick SiO_2_ with the 180 nm thick bottom electrode of Al. The etching time for 15 nm of SiO_2_ is 1 min. Using the chemical selectivity of CF_4_ to etch SiO_2_, we etch two times longer (2 min) in order to form a good undercut in SiO_2_, which is important for the following deposition steps.

**Figure 5:**
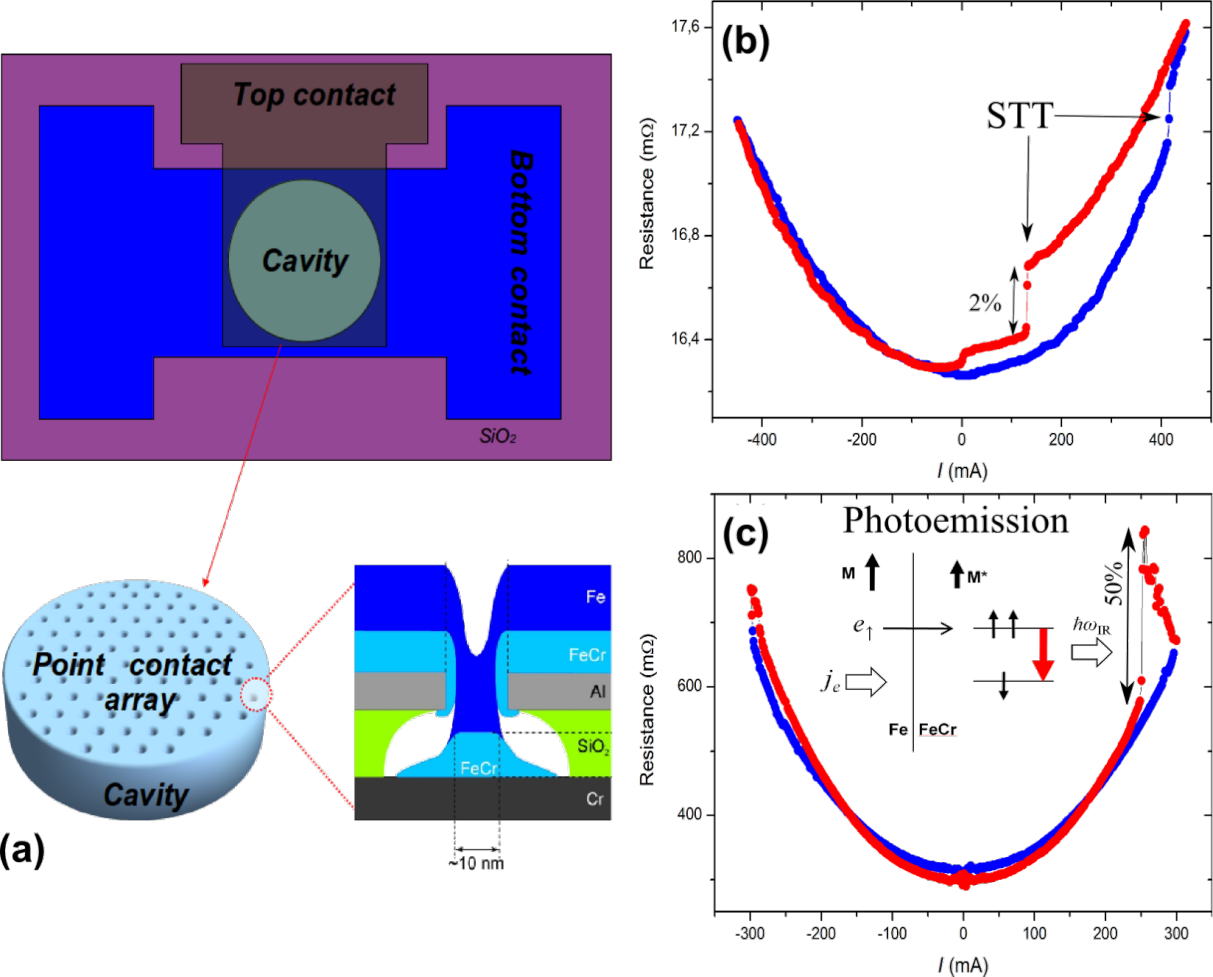
(a) ) Illustration of the integrated circuit device produced by using a colloidal nanoarray mask and three steps of photo-lithography for the bottom electrode, top electrode, and the resonator. Top panel shows the layout of the device, with the top electrode formed in the shape of a photon resonator for IR–terahertz, 10–50 µm diameters. Bottom panel shows the nano-array mask and the core structure of a single sub-10 nm point contact. (b) Resistance versus bias-current characteristic for a Fe/Fe_0.7_Cr_0.3_ point-contact array (low resistance range, 10–20 mΩ), showing current-induced hysteretic switching superposed on to a parabolic phonon background. (c) Resistance versus bias-current for a 36 µm diameter spin-flip laser array (high-resistance limit, ≈1 Ω), showing giant excitations of the threshold type at critical pumping, consistent with the onset of the expected stimulated emission in the device [15]. Red and blue represent up and down sweeps in bias. More details on these measurements can be found in [26].

Magnetron sputtering was used for deposition of the active point-contact region. The material combination was selected to represent the spin-injection laser device [15,24–25], and consisted of a spin-majority/minority ferromagnetic bi-layer Fe_0.7_Cr_0.3_(10 nm)/Fe(15 nm) [25] capped with Cu(10 nm), for spin-population-inversion injection. Even though the deposition technique is not highly directional, we find that the angle of incidence through the 10 nm openings in the mask (angle between the normal to the sample surface and the normal to the sputtering target surface) is an important parameter determining the size of the resulting point contacts: the closer to normal incidence the closer the resulting contact size to the mask feature size (normal incidence), and the larger the angle of incidence the deeper below 10 nm the nanocontacts are due to the double shadowing effect illustrated in Figure 5a (direct mask shadowing and shadowing from material build up on the mask edge). We note that the SiO_2_ under the openings in the Al mask is significantly undercut (approximately 30 nm diameter) and therefore is no obstacle to material deposition into the nanopores. We additionally note that the Al layer, originally ≈15 nm thick, is thinned by the Ar-etching steps to ≈10 nm. Thus, the effective nanopores are an array of ≈10 nm holes in ≈10 nm thin Al.

We used two angles of deposition, near-normal incidence and approximately 45° incidence, and estimate that the average size of the nanocontacts obtained for the angled deposition was approximately 5 nm. Finally, surface protection for subsequent processing steps was done with two layers of Cr(5 nm)/Au(10 nm) deposited by e-beam evaporation. The key elements of the point-contact structure obtained are illustrated in the bottom panel of Figure 5a. The final integration step is photolithographic patterning of the top electrode, in the case of the demonstrator devices below in the shape of a photon resonator for IR–terahertz photons (Figure 5a, top panel; see Experimental for process details).

### Device examples

The focus of this paper is the new method of integrating sub-10 nm structures into nanodevices. We briefly demonstrate the method using two physical effects found in magnetic nanocontacts, namely, spin-magnon and spin-photon relaxation. The method is not limited to photonics or spintronics, however, and should have a wide application range in various types of physical systems.

We first estimate the expected circuit characteristics of our typical integrated point-contact array. For individual 5–10 nm metallic point contacts the resistance is essentially given by the geometry (the so-called Sharvin resistance [27]) and is approximately 10–20 Ω. For an ideal 10 × 10 μm^2^ point-contact array with a 200 nm intercontact spacing, the number of contacts is 2500. Therefore, the expected resistance of the array is on the order of 10 mΩ. A nonideal array would have fewer contacts and therefore higher resistance. Overetching the polystyrene monolayer and sharp-angle deposition of the core material, as discussed in detail above, can result in only a fraction of the array actually being connected and the individual nanocontact size being substantially smaller than the mask feature size of 10 nm, as measured by SEM and AFM. In this limit we are able to reach the array resistance range of the order of 1 Ω.

We have prepared test samples with the point-contact core made of a single nonmagnetic metallic element (Cu with a Cr under-layer), where no effects due to spin-flip relaxation are expected, only phonon relaxation (heat). The typical array resistance is measured to be 10–20 mΩ. The current–voltage characteristic is smooth and approximately parabolic, typical of the expected phonon background. Thus, these test data agreed with the expected behaviour and showed that the fabricated nanocontact arrays are of high quality.

We next demonstrate a point-contact array with the contacts having a magnetic core. More specifically, the core material is a majority/minority ferromagnetic bi-layer of Fe/Fe_0.7_Cr_0.3_ [28], where due to the opposite spin-polarizations of the two materials at the interface a strong spin accumulation is expected. Figure 5b is a resistance versus bias-current characteristic for the device and shows a clear current-induced hysteretic switching, typical of magnetic point contacts [29–30], superposed on the phonon background. The mechanism behind this is the formation of atomic/nanoscale domain walls in the nanoconstriction under the spin-transfer torque (STT) from the spin accumulation at the Fe/FeCr interface. The switching in both directions occurs at one bias polarity, which is characteristic of the STT effect. The change in resistance is approximately 2%, typical of domain wall magnetoresistance. The array resistance is on the order of 10 mΩ, consistent with the expected range for a nearly fully connected sub-10 nm point-contact array. Thus, we demonstrate the STT effect in the fabricated nanodevices. The extremely regular array layout with the extremely small contact size, as well as the relative ease of the colloidal monolayer process, should make this structure very promising for gigahertz nano-oscillators [9–14]. Optimization would involve substituting improved spin-valve materials for the core region and, if needed, tuning the array lattice spacing to achieve better interference of spin-wave modes.

Another interesting application of the spin-majority/minority Fe/Fe_0.7_Cr_0.3_ contact-core material used above is the spin-flip photon-emission effect [15,24–25], which requires spin-polarized and energetically nonequilibrium injection and, therefore, near-ballistic point-contact arrays. The energetics of the process is illustrated in the inset to Figure 5c, where the spin-majority carriers from the Fe injector create spin-population inversion in the spin-minority FeCr, relaxing through emission of terahertz or infrared (IR) photons. To achieve this we have performed fabrication at the limit of the etching and angle-deposition parameters discussed above, for deep sub-10 nm point-contact size and a smaller operating fraction of the array, so that the injection voltage per contact is greater than the exchange splitting in the ferromagnetic point contact core (10–100 mV, see [24–25] for more details). Such high-bias, high-density, spin-polarized injection produces large nonequilibrium spin accumulation in the contact core, which allows spin-flip photon-emission transitions, vertical in the momentum of the electron. A photon emitted by a spin-flip process is contained within the resonator and enhanced by the high-dielectric constant, high-transparency SiO_2_ oxide matrix [15]. The lifetime of the emitted photon is long due to the high transparency of the oxide, so the photon has a high probability to stimulate another spin-flip transition. At a critical bias, a cascading avalanche of stimulated spin-flip photon-emitting transitions, i.e., a laser action, takes place, generating high-density electromagnetic modes in the resonator. This critical photon-emission threshold must manifest itself as threshold-type changes in the current–voltage characteristics of our fully enclosed optical resonator. Such threshold-type excitations, of giant magnitude, are indeed observed in the device resistance (conductance changes of a factor of 2), as shown in Figure 5c. This demonstration opens the way to explore a new type of IR–terahertz lasers based on stimulated spin-flip photon emission [26].

## Conclusion

Colloidal patterning in the form of large-area hexagonal-lattice arrays is demonstrated to scale down to sub-10 nm dimensions in the feature size. This is comparable or smaller than the key relaxation lengths in various materials including metals, which enables a wide range of new applications in nanotechnology. Large-area, near-ballistic-injection point-contact arrays are used to demonstrate integration of the developed nanofabrication technique into new types of spintronic and photonic devices.

## Experimental

**Self-assembled monolayer process sequence:** The first step of the spinning sequence allowed the particles to gently segregate onto the substrate and, to a large extent already here, form a hexagonal pattern. The second step of spinning at a higher speed (rpm) prevented formation of additional layers of vertically stacked particles. The last step was used to remove the remaining solution, predominantly in the corners of the sample. The spinning process was developed by using SiO_2_ substrates, about 2 × 2 cm^2^, covered with a 10 nm thick Au layer. Prior to spinning, the substrate was etched in plasma oxygen for two minutes in order to make the surface hydrophilic [31]. As an alternative route, we found that a good quality monolayer, with a well-defined long-range order, can be obtained if a small amount of Triton X surfactant [32] is added into the colloidal solution. However, the subsequent extensive tests showed that the use of Triton X is problematic for later processing during the device-integration steps. Traces of Triton remaining on the surface produce residue that prevents reliable integration. We therefore selected the route of forming large-area self-assembled monolayer arrays of nanoparticles by making the substrate hydrophilic with the help of RIE plasma oxygen. The equipment specifics are Oxford Plasmalab 100, with the capability for gases O_2_, Ar, CF_4_, CHF_3_, SF_6_, Cl_2_.

**Colloidal mask transfer:** Oxygen plasma was tested for removal of the particles, without success. Chemical removal of polystyrene particles after down-scaling by using acetone and mechanical polishing also did not work. Another technique tested with only partial success was heating of the sample just below the melting point of polystyrene, where the dilatation coefficient makes the particles expand in volume significantly and thereby break open holes in the Al film. This is a promising technique; however, we found it difficult to control the size and shape of the resulting ≈10 nm holes opened by polystyrene exploding through the Al.

**Optical lithography:** A double-layer resist LOR7B(500 nm)/S1813(1.5 µm) is spun onto the Au surface, thermally treated, mask exposed, developed, ion milled for 2 h, and lifted off in 1165 remover at 60 °C to form a 100 µm wide bottom electrode.

**Optical lithography of the top electrode/resonator:** The process is analogous to the one used for the bottom electrode, but employs a different photomask. The top electrode mask has different diameter disks and half-disks in the range of 10–50 µm. The pattern transfer is done by ion milling for 1 h. The etching time was calibrated by using surface profilometry such as to stop the etching at the Al bottom electrode. The sample was then capped with a 40 nm thick SiO_2_ layer for insulation, rotating the sample holder during deposition. Finally, the resist was lifted off, and the last step of lithography was the use of negative resist and deposition of a 200 nm thick Al top electrode.

**Transport measurements:** The current–voltage (*I*–*V*) characteristics of the integrated devices were measured by the conventional four-point technique, with the device resistance defined as *R*(*I*) = *V*(*I*)/*I*. For more details on the spin-transfer-torque measurements see [29–30]. For more details on stimulated spin-photo-emission (spin lasing) see [15,24–26].
